# Manchester's Milk Supply

**Published:** 1920-04-24

**Authors:** 


### Manchester's Milk Supply.

The Manchester proposal for a municipal milk supply is hanging fire. When it last came before the City Council, the Public Health Committee reported that the existing state of affairs was unsatisfactory, and could only be remedied by proper organisation and control. If the Corporation adopted the best plan—namely, the acquisition of rights of distribution, at present held by dealers and farmers—the compensation payable would amount to £460,000. The Committee estimated that the annual charges on this could be met out of the profits under the scheme. The Finance Committee, on the other hand, was apparently rather nervous about the plan, and feared that outlay would be required for the establishment of collecting and distributing centres and dairies. They therefore reported against the scheme in view of the many other obligations involving capital expenditure, such as housing, and the Council deferred the matter for the purpose of securing more information and more time for discussion.

